# What Our Dairy Cattle Inherit1Read at the annual meeting of the Pennsylvania State Veterinary Medical Association, March, 1898.

**Published:** 1898-04

**Authors:** M. E. Conard

**Affiliations:** West Grove, Pa.


### WHAT OUR DAIRY CATTLE INHERIT.[1]

By M. E. Conard, V.M.D.,

WEST GROVE, PA.

Inheritance, as the term is generally used, means something willed, bequeathed, or given by one person to another of a younger generation, as money, bonds, lands, or something representing wealth along financial lines; but as our dairy-cattle do not indulge in the use of the filthy lucre, with its innumerable concomitant evils, we must consider their inheritance from a broader and more comprehensive standpoint. Scientifically or physiologically speaking, an inheritance is a trait of character or anatomical conformation given to an individual by the presence of a like condition in one or both of its parents. (Like begets like.) The individual characteristics of shape, size, conformation, character, or mental development, of which we as a human race are so proud, are simply the result of transmission or inheritance, if you please, and are modified, multiplied, transposed, or deviated by the conditions of life to which we are subjected, developing, as it were, to meet the demands of our environments, but not adding one new or original trait of character to our being. In short, we are made up of second-hand characteristics, some of which, for the want of proper surroundings or circumstances, may have remained almost or wholly dormant in our parents, but were nevertheless present, and when we are surrounded by conditions suitable for their development they predominate over those apparent originalities of the parent which are in reality prominent individualities, resulting in an excessive development in certain lines.

Now the same general rule must apply to the lower order of this world's inhabitants that governs the development of "God's image." Then, let us consider the primitive condition, the subsequent environment, and the evolutionary process through which our dairy-cattle have passed to become the wet-nurse of the human race, and note the result.

The original cow, the female of the bovine race, was at first possessed with the responsibility of reproducing her species, and producing sufficient milk for its sustenance for the first few weeks of its life; then lactation ended, the cow becoming pregnant again, and repeating the process annually, giving the system ample time

[1] Read at the annual meeting of the Pennsylvania State Veterinary Medical Association, March, 1898.

to regain its normal vigor and strength before the advent of another birth.

But when applied to the use of man for dairy purposes, what was required of her, and what were her environments? She was required to convert the greatest possible amount of feed into marketable products, and as her life-value was and is measured only by her ability to produce milk and butter, the process of lactation was steadily increased to its utmost capacity, often being terminated only by death, having been occasionally interrupted by the birth of a poor, weakly, little, half-starved calf; and this is the No. 1. dairy-cow of the present day.

You may ask how this change has been brought about, and who is responsible. A moment of reflection will show you. It is simply the result of the transmission of paternal characteristics and impressions, which is the sole object of careful breeding. How is it done, do you ask? By so feeding, housing, nursing, milking, and generally treating a cow as to develop her milk-producing organs away out of proportion to and in excess of the rest of her physical structure, and to prolong the period of lactation until the birth of the calf, which she is expected to create after her own image.

Now you will all agree with me that a pregnant cow must necessarily, according to natural laws, emphasize in her offspring the character or quality that is made the most prominent in her being during her term of pregnancy; and what is more prominent than the production of milk? Now if this calf should be a heifer, she is carefully raised and mated with a male that has been produced under like conditions; possibly a descendant of the same ancestry, who has had instilled into him the property of begetting the same characteristics. The result is an unbalanced accumulation of forces in the next generation, with an insufficient supply of vitality to run the machine to its proper capacity, and the result must be a breakdown.

This line of breeding, developing, transmitting, and inheriting only those properties that seem to apply to our immediate needs or demands in the production of milk, butter, or cheese, regardless of the constitution and vigor of the race, must of necessity, if followed generation after generation, have the effect to detract from the vital energy the powers of resisting disease, the ability to produce a strong, healthy offspring. It must also create a tendency in generations to come, or a predisposition, if you please, to such diseases or ailments as tuberculosis, abortion, parturient apoplexy, or such others as may come in way. For as any set of organs are developed at the expense of another set, so the opportunity is offered for the entrance of such as the robbed and weakened are heir to. Do not understand me to be opposed to good breeding. I am far from it. But good breeding of dairy-cattle, as well as any other kind of live-stock, or even the human race, demands an equal proportionate development of each and every part, so that there can be no jostling or discord. No diverting of vitality out of normal channels. In short, she must be so constituted that she can produce a profitable amount of marketable products, and not break down in middle life. She must also produce a good strong calf every year. An animal of this kind is not so likely to become a medium in which the ever-present germs of disease find favorable soil for their growth, multiplication, and perpetuation, and through which they can find their way into the stomachs of babies and convalescents.

Now there is not a man in this room, I trust, who would not speak in the most commendatory terms of the most excellent work being done toward the eradication of diseases of live-stock in this grand old Commonwealth by our Live-stock Sanitary Board, under the efficient direction of our mutual friend and brother, Dr. Leonard Pearson. I wish to say, in conclusion, that it is our moral duty as custodians of the health of the live-stock in our respective districts so to influence the breeding, care, and management of our live-stock, particularly dairy-cattle, that we may assist in the good work by producing a race of cattle that have not inherited the ills of the present generation, but by their vigorous constitutions are almost immune from disease.

